# EMT-Mediated Acquired EGFR-TKI Resistance in NSCLC: Mechanisms and Strategies

**DOI:** 10.3389/fonc.2019.01044

**Published:** 2019-10-11

**Authors:** Xuan Zhu, Lijie Chen, Ling Liu, Xing Niu

**Affiliations:** ^1^Institute of Translational Medicine, China Medical University, Shenyang, China; ^2^Department of Surgery, First Affiliated Hospital of China Medical University, Shenyang, China; ^3^Department of Third Clinical College, China Medical University, Shenyang, China; ^4^Department of College of Stomatology, China Medical University, Shenyang, China; ^5^Department of Second Clinical College, Shengjing Hospital Affiliated to China Medical University, Shenyang, China

**Keywords:** epithelial-mesenchymal transition, acquired resistance, therapeutic strategies, non-small-cell lung cancer, epidermal growth factor receptor tyrosine kinase inhibitors

## Abstract

Acquired resistance inevitably limits the curative effects of epidermal growth factor receptor tyrosine kinase inhibitors (EGFR-TKIs), which represent the classical paradigm of molecular-targeted therapies in non-small-cell lung cancer (NSCLC). How to break such a bottleneck becomes a pressing problem in cancer treatment. The epithelial-mesenchymal transition (EMT) is a dynamic process that governs biological changes in various aspects of malignancies, notably drug resistance. Progress in delineating the nature of this process offers an opportunity to develop clinical therapeutics to tackle resistance toward anticancer agents. Herein, we seek to provide a framework for the mechanistic underpinnings on the EMT-mediated acquisition of EGFR-TKI resistance, with a focus on NSCLC, and raise the question of what therapeutic strategies along this line should be pursued to optimize the efficacy in clinical practice.

## Introduction

Lung cancer is the leading cause of cancer mortality among males worldwide and females in more developed countries (1). Non-small-cell lung cancer (NSCLC) accounts for 85% of cases (2). Treatment with epidermal growth factor receptor tyrosine kinase inhibitors (EGFR-TKIs) has been the first-line treatment for NSCLC patients harboring activating EGFR mutations (3, 4). Nonetheless, an overwhelming majority of patients who initially respond to EGFR-TKIs treatment eventually develop acquired resistance, which invariably limits the clinical efficacy of therapy. Epithelial-mesenchymal transition (EMT) has long been linked to acquired EGFR-TKI resistance in NSCLC, but mechanisms underlying EMT-dependent acquisition of EGFR-TKIs resistance are still far from fully explored. EMT, a reversible biological process, wherein cells undergo a switch from epithelial phenotype to mesenchymal state. EMT is involved in both physiological and pathological processes. Originally described in the context of embryonic development, to date, EMT has been correlated closely with tumorigenesis, invasion, metastasis and drug resistance (5–7). Due to the reversibility of EMT process, further understanding of the intricate relationship between EMT and NSCLC, particularly the mechanistic basis responsible for EMT-mediated resistance contributes to improving the benefit of TKI treatment for NSCLC patients. In this review, we focus on potential mechanisms of acquired EGFR-TKI resistance induced by EMT and discuss promising avenues for targeting the EMT program as a strategy for NSCLC treatment.

## EMT and Drug Resistance

EMT is a process involved profound changes in both morphology and physiology (6). During EMT, cells downregulate the expression of epithelial proteins containing E-cadherin, whereas upregulate the expression of mesenchymal proteins, such as N-cadherin and vimentin. In addition, cells undergoing EMT are characterized by the loss of apico-basal polarity and intact cell-cell junctions, while acquisition of front-rear polarity and dramatic remodeling of the cytoskeleton (8). Also, EMT program endows cells with enhanced invasive capacity, therapeutic resistance and cancer stem-cell-like properties (7, 9). These findings have inspired the interests in EMT in the cancer field during the last decade. Resistance to EGFR-TKI represents a prime obstacle for NSCLC treatment, making it of particular importance to delve into the detailed mechanisms. Hence, how the execution of EMT contributes to drug resistance has been studied extensively.

### EMT Phenotype Attributes in EGFR-TKI Resistant NSCLC Cells

A growing number of studies point to a molecular association between EMT and drug resistance. Some clinical and molecular evidences were established in the early 2010s. A lung cancer patient who developed acquired resistance to erlotinib was reported to be found EMT in the tissue sample. It is worth noting that there were no other known resistant mechanisms including T790M mutation and MET amplification. In an additional experiment, the gefitinib-resistant subline of HCC827 cells shows phenotypic and molecular changes that are consistent with EMT (10). Emerging evidence agrees that gefitinib-resistance PC9 and HCC827 cells convert to a mesenchymal phenotype. Accompanied by the decreased expression of E-cadherin, the expression of N-cadherin and other mesenchymal markers is elevated, illustrating the emergency of EMT (11). Furthermore, NSCLC cells with acquired resistance to gefitinib or osimertinib (AZD9291) showed EMT characteristics, with a decrease in E-cadherin, and increases in vimentin and stemness, without any EGFR secondary mutations (12). Taken together, it is not surprising that EMT is considered as one of the possible mechanisms for the acquired resistance to EGFR-TKIs in NSCLC.

### Role of EMT in Resistance to Targeted Therapy

Accumulating evidence has highlighted that activation of Notch signaling participates in EMT in NSCLC (13). Earlier studies discovered that aberrant Notch-1 signaling leads to acquired resistance to EGFR-TKI by triggering EMT and silencing of Notch-1 using small interfering RNA (siRNA) increases the sensitivity of gefitinib (14). The orchestrated changes in gene expression that favors EMT results from acting cooperatively of various master regulators, most notably Snail, Slug, Twist and zinc-finger E-box-binding (ZEB) transcription factors (8). Recent data have proven that the overexpression of Slug and Snail, which are identified as EMT inducers, promotes gefitinib resistance. Remarkably, recovery of TKI sensitivity is associated with the EMT reversion, since blocking the reversal of EMT by the forced expression of EMT inducers can suppress this effect (11). Collectively, these experimental observations reveal the notion that EMT is a vital event in the development of acquired resistance to EGFR-TKIs.

### Mechanisms Associated With EGFR-TKI Resistance via EMT

Despite the vast body of studies regarding the role of EMT in targeted therapy for NSCLC patients, its potential mechanisms are not entirely clear. The mechanisms that govern the EMT are non-linear complex networks (15). Various extracellular signal factor stimuli and the activation of the corresponding intracellular signaling pathway, ultimately results in the downregulation of E-cadherin, which is considered as the hallmark of EMT (8).

### TGF-β: Inducer of EMT

**Figure 1:**
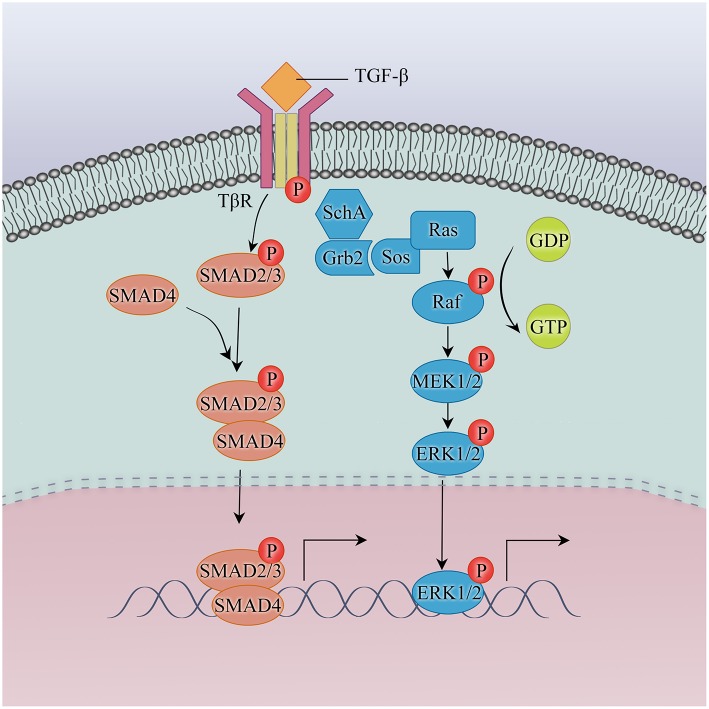
TGF-β/SMAD and non-SMAD pathways in EMT.

Among flexible regulatory networks of EMT, one of the best-characterized inducers is transforming growth factor-β (TGF-β), which has context-dependent effects on cancer progression. TGF-β can be tumor suppressive in pre-malignant epithelial cells by cell cycle arrest and oncogene suppression. But in the context of advanced carcinoma cells, the status of TGF-β is assigned to pro-tumorigenic via mechanisms including promoting tumor angiogenesis, restraining the function of immune system as well as the activation of EMT (16, 17). Acting as a predominant inducer, TGF-β can induce EMT through SMAD-medicated and non-SMAD pathways (Figure 1).

Based on data in cellular and animal models, exposure to gefitinib with increasing concentrations is sufficient to raise the level of TGF-β secretion in HCC4006 and HCC827, going with increased phosphorylation of downstream proteins, SMAD2 and SMAD3. A TGF-β autocrine loop is established by the EGFR inhibition in NSCLC with EGFR mutations, facilitating TGF-β-stimulated activation of SMAD pathway, thus playing a crucial role in EMT (18). How SMAD pathway contributes mechanistically to EMT has been documented. TGF-β family receptor is a heterotetrameric complex with two type I and two type II components, comprising of three distinct regions: an extracellular N-terminal ligand-binding domain, an intracellular C-terminal Ser/Thr kinase domain and centrally located transmembrane domain. Coupling of a ligand with TGF-β receptor complexes, the TβRII (type II) is activated through phosphorylation and thus activates the TβRI (type I), which in turn phosphorylates the SMADs of kinase domain at the C-terminal sequence. In response to receptor-phosphorylated SMAD2 and SMAD3 combination with SMAD4, the trimeric complexes subsequently translocate from cytoplasm to nucleus. Via interacting with the DNA-binding transcription factor, SMAD complexes serve as transcriptional coactivators to activate transcription of mesenchymal genes and transcriptional corepressors to repress transcription of epithelial genes (8).

SMADs also increase the activity of EMT transcription factors as well. ZEB1 is overexpression in HCC4006ER cells which take on mesenchymal characteristics and develop erlotinib resistance by the activation of TGF-β/SMAD pathway. Correspondingly, knockdown of ZEB1 can reverse EMT and restore the sensitivity to TKI. This finding identifies ZEB1 as a remarkable regulator in TGF-β/SMAD-related EMT and TKI resistance (19).

Apart from its role through SMAD pathway, TGF-β activates the MAPK pathway, which also results in EMT-mediated resistance to EGFR-TKI. Blocking ERK signaling by MEK1/2 inhibitor can prevent TGF-β-induced EMT, indicating that MAPK pathway plays a key role in the induction of EMT by TGF-β (5). Upon TGF-β stimulation, the tyrosine and serine phosphorylation of SRC homology 2 domain-containing-transforming A (SchA) is induced, which is mechanistically associated with TβRI. Phosphorylated SchA possesses a growth factor receptor-bound-2 (Grb2) binding site and induces its association with son-of-sevenless (Sos), then converts Ras into active Ras-GTP, thereby initiating the downstream Ras-Raf-MEK1/2-ERK1/2 cascade (20). Of note, enhanced sensitivity to gefitinib is in line with changes in epithelial and mesenchymal markers. After reaching a peak, heightened responses to gefitinib by chronic MEK inhibition eventually decline (5). In light of this, discovering alternative signaling inhibitions that render more stable maintenance of an epithelial phenotype awaits further investigation.

Additionally, crosstalk exists between the TGF-β and Notch signaling to coordinately regulate the reprogramming of gene expression in EMT (21). TGF-β can significantly increase the expression of Notch downstream target genes, namely HEY-1 and HES-1. Consistent with this notion, pharmacological inhibition of Notch by histone deacetylase6 (HDAC6)-targeted siRNA leads to decreased expression of EMT genes, HEY-1 and HES-1, suggesting that HDAC6 is required for Notch activation by TGF-β. Further experiment illuminates HDAC6 is activated then deacetylates its substrate, HSP90, in response to TGF-β stimulation, which is of necessity in the cleavage of Notch receptor. This enables the release of Notch intracellular domain (NICD) and then enter the nucleus, where in cooperation with RBP-J (also known as CSL) as a transcription cofactor to regulate the expression of target genes, including Hey and Hes (22). Given that overexpression of HDAC6 is common in lung adenocarcinoma cell lines and confers resistance to gefitinib, interplay amongst TGF-β, Notch signaling and HDAC6 may be implicated in EMT and resistance to EGFR-TKI (23).

### Induction of IGF1 and EMT

The insulin-like growth factor (IGF) signaling is thought to play a particularly prominent role in growth, development and apoptosis. The insulin-like growth factor-1 receptor (IGF-1R), a key signaling element in the IGF system, is a transmembrane tyrosine kinase receptor, which exerts its effect by binding to the ligand IGF-1. Once activated, IGF-1R is capable of intrinsic tyrosine kinase phosphorylation and activating multiple intracellular adaptor proteins, such as insulin receptor substrate-1 (IRS-1) and Shc to transmit signals, leading to the activation of downstream signaling pathway (24, 25). Recently, various studies have revealed that IGF-1/IGF-1R also appears to take part in EMT and drug resistance (24, 26, 27). NSCLC patients with elevated expression of IGF-1R show poor responses to EGFR-TKIs treatment, strongly suggesting that activation of the IGF-1R pathway is relevant to resistance against EGFR-TKIs (27). However, the importance of IGF-1R-induced EMT in driving the resistance to TKI, specially NSCLC, remains largely obscure.

**Figure 2:**
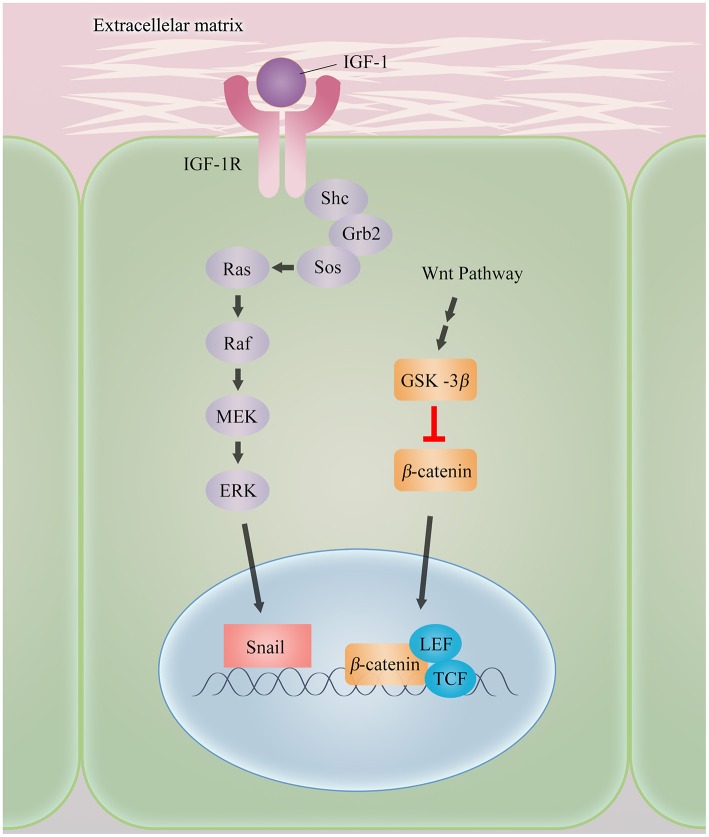
Mechanisms of IGF-1R-induced EMT.

Several works have been undertaken to elucidate the interplay between IGF-1R and TKI resistance (Figure 2). For example, in a model of EGFR-TKI-resistant NSCLC, in which along with IGF-1R upregulation, cells display a highly EMT phenotype. Furthermore, silencing of IGF-1R or overexpression of E-cadherin dramatically represses the EMT program and decreases the cells survival against EGFR-TKI. It should be noted that the expression of Snail and nucleus β-catenin are subsequently enhanced in IGF-1R-dependent EMT. In addition, not PI3k/AKT pathway, but rather the activation of ERK/MAPK signaling is proven to contribute critically to this process in both PC-9 and HC460 cell lines (26). Taken as a whole, these experimental observations provide direct evidence that upregulating of Snail expression arising from activating ERK/MAPK signaling and propelling β-catenin relocation from the cell membrane into nucleus might engender IGF-1R-induced EMT in NSCLC. The synergistic cooperation between these two processes stifles the expression of E-cadherin directly, thereby conferring mesenchymal attributes and acquired resistance to EGFR-TKIs in NSCLC. Of interest, β-catenin is the downstream signal molecule of Wnt signaling. Wnt signaling is considered to promote EMT by impeding the function of glycogen synthase kinase-3β (GSK-3β) to stabilize β-catenin, which translocates to the nucleus and interacts with the transcription factors lymphoid enhancer-binding factor 1 (LEF) and T cell factor (TCF) to define EMT (8). It is increasingly clear that the presence of interaction between the IGF-1R and Wnt/β-catenin in the context of EMT-mediated TKI resistance.

In contrast to the prevailing studies that focus on the impact of specific receptors in resistance, a report has furnished evidence from different viewpoints. Genetic deletion is used to functionally remove IGF-1R in HCC827 cells. Upon exposure to continuous high-dose erlotinib, HCC827 (IGF-1R^−/−^) ER cells do not exhibit an EMT phenotype shift like HCC827ER cells as expected. Intriguingly, acquired resistance is developed by bypass signaling, namely MET-amplification, indicating that IGF-1R is not essential to maintain the gained TKI-resistant state in NSCLC (28). Indeed, results of these data offer us better insights into the resistance by induction of IGF-1R and underscore the complexity of underlying resistance mechanisms, that is under a given TKI selective pressure, cells are prone to find another way to develop resistance in the absence of certain mechanism.

### Key Signaling Pathways in EMT

**Figure 3:**
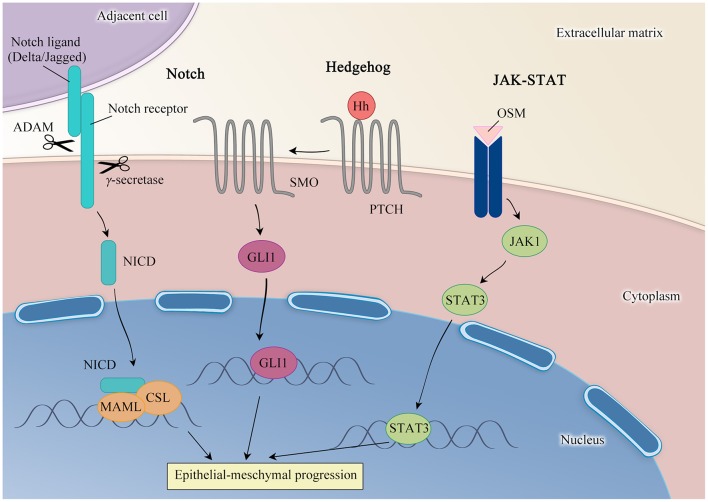
Key signaling pathways involved in EMT.

As with growth factors mentioned above, Notch signaling has been recognized as a potent mediator of EMT (Figure 3). In mammals, there are four receptors (Notch1-Notch4) and two families of Notch ligands, Delta-like (DLL1/3/4) and Jagged-like (JAG1/2) have been identified. Simply speaking, Notch signaling is an intercellular communication mechanism, in which a Notch ligand on the surface of cell induces a suite of proteolytic events including sequential cleavage by ADAM enzymes and then γ-secretase. These events permit the liberation of NICD, which translocates to the nucleus to modulate the expression of Notch target genes in company with CSL (also known as RBP-J) and Mastermind-like protein (MAML) as a result (29, 30).

In a previous experiment, inappropriate activation of Notch1 inhibit gefitinib-induced apoptosis relying on caspase-3 inactivation caused by Notch1-induced EMT, Accordingly, out of balance between apoptosis and anti-apoptosis allows resistance to EGFR-TKI (14). Recently, studies in cell culture have exemplified that chronic treatment of gefitinib dramatically enhances ZEB1 expression, together with the repression of miR-200c. Meanwhile, an EMT state is observed in gefitinib-resistant cells. Conversely, knockdown of Notch-1 enables partially reverse EMT and gefitinib resistance, sharing similarity with the effect of miR-200c. This result implies that Notch1 facilitates EMT to mediate ZEB1-induced TKI resistance. Furthermore, treatment with the γ-secretase inhibitor to block Notch signaling, cells gain growing sensitivity to gefitinib, manifesting the central role of Notch in EGFR-TKI resistance and promising therapeutic value for NSCLC patients with EMT-induced resistance. Nevertheless, consideration of both necessary and sufficient part of NICD to transcriptionally attenuate ERBB3 expression by binding to its promoter directly, inhibition of Notch1 inevitably induces ERBB3, a driver of EGFR-mutated lung cancer cell growth (31). To summarize, these findings support the versatile role of Notch1 in EGFR-mutated cells. Under the circumstance that EGFR is inhibited by TKI treatment, Notch1 results in the acquisition of resistance by triggering EMT. Of particular note, the fact that Notch1-ERBB3 axis functions as a universal regulatory mechanism in NSCLC, provides solid evidence and attractive possibilities for combined targeting of Notch1 and EGFR to treat EMT-driven resistance to EGFR-TKI (31). This result, as well as the prior one, validate the knowledge that Notch signaling is a robust contributor of EMT-mediated TKI resistance.

Outside of Notch cascade, the Hedgehog (Hh) pathway is frequently linked with EMT and dwindling response to EGFR-TKI treatment. There are three HH counterparts have been made clear in human: Sonic hedgehog (SHH), Indian hedgehog (IHH) and Desert hedgehog (DHH). In the absence of HH binding, Patch (PTCH) suppresses the activity of Smoothened (SMO), a member of the G protein-coupled receptor superfamily. Interaction of HH ligands with PTCH initiates the Hh signaling cascade through the relief of its inhibitory effect on SMO, giving rise to activation of GLI1, a zinc-finger transcription factor, to play its part in the nucleus (32, 33).

Mounting evidence demonstrates that dysregulation of this pathway is crucial for EMT-dependent resistance. *In vitro* model of acquired resistance to EGFR-TKIs indicates that HCC827GR cells present a mesenchymal signature accompanied by markedly elevated expression of GLI1 and SMO amplification, implying an involvement of the Hh pathway in the course of EMT. Strikingly, interplay between Hh and MET is described in cell lines and tumor xenografts of nude mice. Depletion of SMO and MET concomitantly enhances gefitinib sensitivity and significantly diminishes the phosphorylation level of MAPK and AKT proteins. In brief, excessive activation of Hh pathway, drives EGFR-TKI resistance owing to EMT induction via SMO amplification and concurrent MET activation. Therefore, the combination of Hh and MET inhibitors may yield powerful antitumor effects in EGFR-mutated NSCLC patients (34).

More recently, further cue to emerging importance of Hh signaling in the induction of EMT derives from a series of studies. For instance, findings of a research show the functional links among Hh signaling, EMT, CSC abundance to the acquired resistance of EGFR-TKI (35). Additional experiment designed to investigate the efficacy and mechanisms of acquired resistance during the sequential treatment with first-, second- and third-generation EGFR-TKIs reveals that activation of the Hh pathway is a common nature that all of three lines share. Different from the preceding result in a model of the first-generation inhibitor, MET hyperactivity is not detected in second- and third-generation resistant models, while SMO activation coexists persistently in diverse treatment (36). In addition, synergic role of AXL and Hh pathway has been reported to mediate resistance to second- and third-generation EGFR-TKIs (36). Given all above, the idea that Hedgehog pathway behaves as a fundamental player for EMT-mediated acquired EGFR-TKIs resistance is little surprising.

Other factors are noted to aid the induction of EMT and cancer progression. Src has been reported to elicit cigarette smoking extract (CSE)-induced EMT and resistance to gefitinib, while N-acetylcysteine (NAC) abrogates the resistance through alleviating Src activation and EMT, providing a clue that simultaneous targeting of EGFR-TKI and Src may help in clinical outcomes in EGFR-mutated NSCLC patients with smoking history (37). Another report indicates that smoking abolishes EGFR-TKI therapeutic effects in NSCLC on account of continuously activating ERK1/2 and AKT pathway downstream of EGFR signaling as well as EMT induction (38). Besides, studies have shown that metformin, a well-known antidiabetic drug, effectively overcomes resistance to EGFR-TKI *in vitro* and *in vivo*, by reversing EMT and suppression of interleukin (IL)-6/signal transducer and activator of transcription 3 (STAT3) pathway (39). Recent advance reveals that anticancer drugs activate IL-6 proinflammatory pathway with upregulation of IL-6 and oncostatin-M (OSM) expression. It is noteworthy that acquired resistance cell lines display genic and morphologic changes, suggesting the occurrence of EMT. In addition to STAT3 activation by autocrine, cocultured with cancer-associated fibroblasts (CAFs) in NSCLC cells in culture also gives rise to inflammation in the tumor microenvironment (TEM) via the secretion of IL-6 and OSM, thus leading to resistance to TKIs. However, this result can be prevented by Janus Kinase1 (JAK1) knockdown. In this context, OSMRs/JAK1/STAT3 axis has been proposed to result in TKI resistance in NSCLC (40).

### Regulation of miRNA on EMT

**Figure 4:**
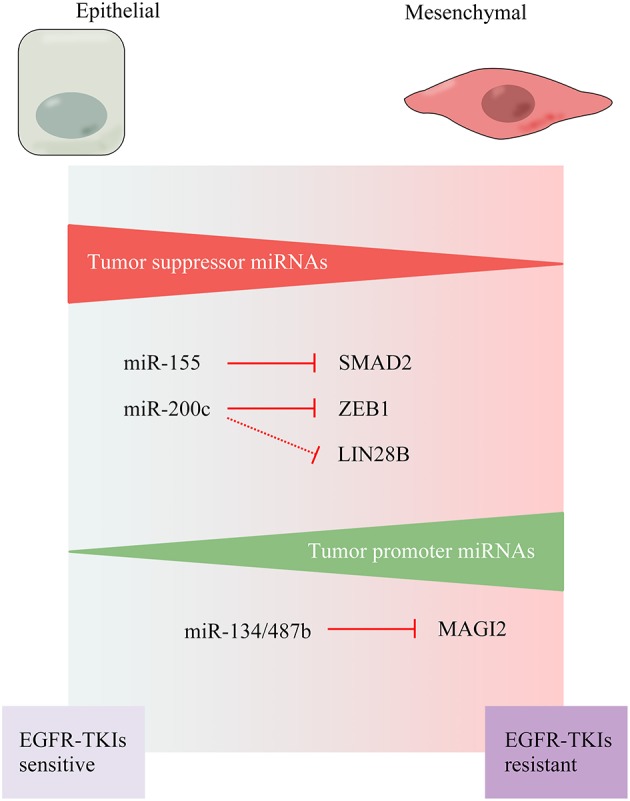
miRNAs regulating EMT program and susceptibility to targeted therapy.

Beyond regulatory networks at the transcriptional level, EMT program is tightly controlled by microRNAs (miRNAs), which has been brought in to focus currently. MiRNAs, small non-coding single-stranded RNAs encompassing 19–25 nucleotides, that modulate gene expression post-transcriptionally, are found to exert pivotal impacts on a variety of biological processes in the development of cancers (41–45). Through coupling with complementary sequences via incompletely base-pairing in the 3′-untranslated region (3′-UTR), miRNAs are able to silence EMT-related molecules, which favor or repress the progress of EMT (Figure 4) (44).

Several lines of evidence shed some light on how the resistance to EGFR-TKI is generated by miRNAs regulation. Experiments in cell lines of NSCLC illuminate that long-term treatment with gefitinib renders changes in miRNAs expression, including depletion of miR-155 and miR-200c. Furthermore, together with the augmented protein level of SMAD2 and ZEB1, the expression of E-cadherin is sharply declined, at least in part, depending on histone modification. In support of this idea, under co-treatment of miR-155 and miR-200c inhibitors, similar results are shown in terms of protein expression and appearance of mesenchymal profile (46). As mentioned, miRNA-200 family members, strong negative regulators of EMT, have drawn substantial attention in tumor biology (47). Analyses of NSCLC cell lines explain that epigenetic modifications, such as promoter hypermethylation, are accounting for the aberrant silencing of miR-200c. Moreover, acquired resistance NSCLC cells bearing the EMT feature exhibit low expression of miR-200c and high LIN28B expression. On the other hand, miR-200c induction not only suppresses the expression of ZEB1, which is known as a mesenchymal marker, but also LIN28B in parallel. Alternatively, knockdown of LIN28B also poses an antitumor effect (48). Overall, the miR-200c/LIN28B axis is complicit in NSCLC resistant against EGFR-TKI. Identification of such a fundamental mechanism involving miR-200c and LIN28B is an ongoing topic of research to pave the way to curative remedy in future.

As is apparent from these instances, miR-200 family members have been depicted as primary miRNA suppressors. For another, tumor promoter miRNAs, provide an additional approach to induce EMT by inhibiting the molecules suppress this step or provoking those initiate it (44). Overexpression of miR-134 and miR-487b as a consequence of TGF-β stimulation has been portrayed to contribute to the occurrence of EMT and drug resistance in NSCLC. Compatible with this result, transfection of anti-miR134/487b inhibits the EMT phenomenon and regains sensitivity to gefitinib likewise. Membrane-associated guanylate kinase, WW, and PDZ domain-containing protein 2 (MAGI2), a scaffold protein is required for phosphatase and tensin homolog (PTEN) stability, is a direct target of miR134 and miR-487b. Reduction of MAGI2 caused by TGF-β leads to PTEN phosphorylation and PI3k/AKT cascade activation. It is therefore promoting the TGF-β-induced EMT and acquired resistance to EGFR-TKI in NSCLC (48).

In summary, numerous studies on the regulation of EMT process by miRNAs unravel their functional roles as tumor suppressors or tumor promoters. MiRNAs seem to participate in carcinoma progression by targeting EMT-related mRNAs in elaborate regulatory networks at the post-transcriptional level, imbuing an additional layer of gene expression. Consequently, these miRNAs are likely to dictate how activation of signaling pathways affects cell fate and determinate the response to targeted agents. Despite the research advances concerning molecular mechanisms of miRNAs in the regulation of EMT in cancers, little is known about its relative contribution on EMT-mediated acquisition of EGFR-TKI resistance, especially in NSCLC. Future effort is needed to fill these gaps for improvements in therapy.

### Relationship Between EMT and CSC

As discussed previously, the conversion of EMT is a key component in malignancy, resulting in disease progression and drug resistance. Cancer stem-like cells (CSCs) are another strong driving force in the development of therapeutic resistance, ranging from conventional chemotherapeutics to molecularly targeted therapy (49, 50). The CSC paradigm postulates the existence of a minor subpopulation of neoplastic cells that hold self-renewal ability to generate daughter CSCs and differentiation potency to produce non-CSCs progenies. Implicit in this paradigm is the capacity of seeding new tumors and the generation of tumor heterogeneity (51).

Currently available evidence points to the mutual relationship between these two phenotypes in EGFR-TKI resistance. One example is the identification of EMT features and stem cell-like properties in a study based upon afatinib-resistant cell lines, revealing a possible association in the midst of EMT, stem cell-like signature and EGFR-TKI resistance (52). Data from a model with ectopic activation of Hh pathway, NSCLC cells exhibit EMT attributes and ABCG2 upregulation. ABCG2, have been referred as a stem cell marker, severs as multidrug resistance pumps to EGFR-TKIs, which is controlled by Hh signaling directly. Blocking Hh pathway by a specific inhibitor, NSCLC cells become able to resensitize to EGFR-TKI, along with the reversal of EMT phenotype and CSCs reduction (35). In addition, analyses of the molecular characteristics of gefitinib-resistance chemokine receptor 4 (CXCR4)-positive cells have unveiled that upregulation of CXCR4, to some degree, is induced by EMT. Given the knowledge that CXCR4-positive cells have the property of stemness, it is conceivable that CXCR4 is a hub to find the connection between EMT and CSCs, both of which correlate intimately with EGFR-TKI resistance (53). More recently, results of *in-vitro* assays and murine model, have reinforced the view that TKI treatment triggers EMT and confers a CSC phenotype in NSCLC cells, subsequently contributing to drug resistance. Further exploration reveals that the imperative character of AKT/FOXM1/stathmin axis in TKI-induced CSC enrichment and drug resistance, which is verified by the assessment of EMT and CSC biomarkers. Importantly, genetic manipulation of FOXM1 and stathmin 1, or blockade of PI3k/AKT pathway may impair CSC abundance and improve the response toward TKI agents (9).

Taking these into account, the connection between EMT and a CSC phenotype is proposed by massive experimental data, the mechanistic basis within these two events is unsolved yet. Since EMT appears to be a crucial strategy employed by tumor cells to acquire CSC-like properties, which are coupled to promote the potential of resisting to antitumor drugs, making EMT an appealing biological target for cancer therapy. Thus, a surge of attention has been given to targeting EMT regulators to eradicate CSCs (54). As such, whether EMT program is a necessary or sufficient condition for the enrichment of CSCs, and what is the relationship as well as the distinction between EMT and CSCs remains to be addressed. Given that CSCs are subjected to robust regulation by tumor microenvironment, one possible explanation is that alternations in diverse factors induced by carcinoma cells undergoing EMT change surrounding microenvironment, which affects the induction and maintenance of CSCs (55).

## Strategies to Overcome EMT-Dependent Acquisition of EGFR-TKI Resistance

Drug resistance is a pervasive barrier in TKI therapy. Unremitting studies enable us to better understand its biological underpinnings in lung cancer and lend added insights into clinical implications, such as novel therapeutic strategies to combat TKI resistance (56). EMT, a well-coordinated process, has been viewed as a major mechanism of EGFR-TKI resistance in NSCLC, in this setting, strategies aimed at extracellular stimuli and intracellular signaling pathways related to EMT are rapidly accumulating (57). In this section, we recapitulate the emerging agents that impinging on EMT-associated signaling networks in the field of therapeutic intervention for NSCLC (Table 1).

**Table 1:** Overview of clinical data involving EMT-related pathway inhibition in NSCLC.

**Mechanism of drug action**	**Drugs**	**Phase**	**Tumor type**	**Preliminary results**	**References**
**TGF-β** **signaling inhibitors**					
TGF-β kinase inhibitor	Galunisertib (LY2157299)	I	Solid tumors	An acceptable tolerability and safety profile	(61)
	Galunisertib (LY2157299)	I	Glioma and solid tumors	No medically relevant cardiac toxicity detected	(62)
Tumor cell vaccine	Belagenpumatucel-L	III	NSCLC	Good tolerability, no serious safety issues, no improvement in patients' survival after platinum-based chemotherapy	(63)
**IGF-1R signaling inhibitors**					
IGF-1R mAb	Figitumumab	III	NSCLC	No improvement in patients' survival after figitumumab plus chemotherapy	(65)
	Figitumumab	III	NSCLC	No improvement in patients' survival after erlotinib plus figitumumab	(66)
IGF-1R/INSR kinase inhibitor	Linsitinib (OSI-906)	II	NSCLC	A poor patients' prognosis caused by linsitinib plus erlotinib	(67)
	Linsitinib (OSI-906)	II	NSCLC	No improvement in patients' survival after linsitinib plus erlotinib	(68)
IGF-1R kinase inhibitor	AXL1717	I	NSCLC	Bone marrow toxicity profile	(69)
	AXL1717	II	NSCLC	Low incidence of grade 3/4 neutropenia	(70)
**RAS-RAF-MAPK inhibitors**					
BAF inhibitor + MEK inhibitor	Dabrafenib + Trametinib	II	NSCLC	Clinically meaningful antitumour activity and a manageable safety profile	(71)
**PI3k-AKT-mTOR inhibitors**					
PI3k inhibitor	Pictilisib (GDC-0941)	I	Solid tumors/NSCLC	Well-toleration and preliminary antitumor activity	(72–74)
mTOR inhibitor	Everolimus	I	NSCLC	Measurable, dose-dependent, biologic, metabolic, and antitumor activity of everolimus in early-stage NSCLC	(75)
AKT inhibitor	MK-2206	I	Solid tumors	Well-toleration and preliminary antitumor activity for MK-2206 plus carboplatin and paclitaxel, docetaxel, or erlotinib	(76)
**Notch signaling inhibitors**					
Γ-secretase inhibitor (GSI)	PF-03084014	I	Solid tumors	Well-toleration and a dose-dependent pharmacokinetic profile	(77)
	RO4929097	I	Solid tumors	Autoinduction at all dose levels that limited the ability to dose escalate the doses	(78)
	BMS-906024	Preclinical	Leukemia and solid tumors	–	(79)
	LY900009	I	Advanced cancer	Recommended maximum tolerated dose at 30 mg/kg Q3W	(80)
DLL4 mAb	Enoticumab (REGN421)	I	Solid tumors	Recommended phase II dose of 4 mg/kg Q3W and 3 mg/kg Q2W	(81)
	Demcizumab	I	NSCLC	Identification of a truncated dosing regimen and recommended phase II dose of demcizumab (5 mg/kg q3-weekly ×4)	(82)
**Hedgehog signaling inhibitors**					
SMO inhibitor	PF-04449913	I	Solid tumors	Maximum tolerated dose at 320 mg/day, with preliminary antitumor activity	(84)
	TAK-441	I	Solid tumors	Maximum tolerated dose at 1,600 mg/day, with preliminary antitumor activity	(85)
	Sonidegib (LDE225)	I	Solid tumors	Tolerance differences between East Asian and Western populations	(86)
**Other inhibitors**					
IL-6 inhibitor	Siltuximab	I	Solid tumors	Well-tolerated but no clinical activity in solid tumors	(89)
STAT3 inhibitor	AZD9150	I	Lymphoma and lung cancer	AZD9150 preclinical activity translated into single-agent antitumor activity	(90)
	OPB-51602	I	Solid tumors	A longer half-life and poorer tolerability of continuous dosing compared with intermittent dosing	(91)
HDAC inhibitor	Panobinostat (LBH589)	I	NSCLC and head-and-neck cancer	Maximum tolerated dose at 30 mg (panobinostat) and 100 mg (erlotinib)	(95)
	Romidepsin	I	NSCLC	A well-tolerability and effects on relevant molecular targets	(96)

### Targeting TGF-β

TGF-β can foster cancer progression by stimulating EMT, identification of its pro-oncogenic role has provided a rationale for the development of drugs targeting TGF-β in cancer treatment. However, the pleiotropic functions of TGF-β pose a formidable hurdle for the application of this strategy (58). Small molecule inhibitors (SMIs) embody a spectrum of pharmacological approaches to reversing EMT by blockage of TGF-β signaling, among them galunisertib (LY2157299) is the most well-studied one (59). It had an acceptable safety profile and achieved success with a manner of intermittent dosing regimen based on the preclinical pharmacokinetic/pharmacodynamic models and predictive biomarkers development in a first-in-human dose (FHD) study in glioma patients (60). Given the encouraging results of the FHD study, a nonrandomized, open-label phase I clinical trial was conducted in Asian populations, 12 advanced solid tumors Japanese patients were treated with the improved dosage in lack of cardiotoxicity or other dose-limiting toxicities during the treatment of galunisertib (61). Recently, a phase 2 study demonstrated that galunisertib plus sorafenib showed acceptable safety and a prolonged overall survival outcome for advanced hepatocellular carcinoma (62). Owing to cardiac toxicities in animals, the implementation of comprehensive cardiac monitoring throughout the course of the FHD study becomes an urgent imperative. Yet, administration of LY2157299 did not observe significant cardiac toxicity in either a short or long therapeutic procedure manner, thereby providing support for advancing clinical development (63).

Another avenue for blocking this pathway is immunotherapy. Belagenpumatucel-L, an allogeneic tumor cell vaccine, was evaluated in a randomized, placebo-controlled phase III study in III/IV NSCLC patients. Results in this work showed that it was well tolerated and rendered better survival compared to the placebo cohort. Unsatisfactorily, patients who completed the previous chemotherapy more than 12 weeks failed to receive any advantages. The clinical utility of belagenpumatucel-L merits further evaluation as a consequence (64).

### Targeting IGF-1R

IGF-1R pathway also promotes EMT and evolution of resistance against TKI in clinical practice, hence IGF-1R pathway has evolved into an important target across different malignancies. Therapeutic agents studied in clinical trials involving patients with NSCLC include both monoclonal antibodies (mAbs) to IGF-1R and small molecule tyrosine kinase inhibitor of IGF-1R (65). Two phase III trials assessed the combination of figitumumab with cytotoxic chemotherapy (paclitaxel and carboplatin) and EGFR-TKI (erlotinib) in patients with advanced NSCLC were closed early with disappointing consequences (66, 67). The all-causality serious adverse events (SAEs) and treatment-related deaths in a subset of patients receiving figitumumab plus carboplatin/paclitaxel, as compared with those receiving chemotherapy alone, witnessed the failure of this clinical trial (67). Similarly, figitumumab combined with erlotinib did not hold superiority over erlotinib alone in another phase III study (66). Whereas these unimpressive outcomes in the clinic, further clinical development of figitumumab has been halted prematurely. However, in a recent phase II trial, chemotherapy-naïve patients harboring activating EGFR mutations with stage IIIB/IV or post-surgical recurrent non-squamous NSCLC were treated with erlotinib induction at 150 mg/day for 3 months, followed by cytotoxic chemotherapy with platinum plus pemetrexed, with or without bevacizumab. Preliminary results revealed that the therapy was well tolerated and may be a treatment option for patients responsive to short-term erlotinib treatment (68). In addition, Linsitinib (OSI-906) is an orally bioavailable, dual small-molecule inhibitor of IGF-1R and insulin receptor (INSR), whose efficacy is currently being tested in clinical studies. A randomized phase II study of the addition of linsitinib to erlotinib in chemotherapy-naive NSCLC patients with positive EGFR mutation demonstrated a detrimental effect that led to inferior efficacy (69). This is not the only clinical trial of linsitinib failing to match the expected results in concord with preclinical data. In a phase II trial, linsitinib maintenance therapy in conjunction with erlotinib showed no difference in progression-free survival (PFS) and overall survival (OS) in NSCLC patients without progression following platinum-based regimen (70). AXL1717 is another small molecular agent which can modulate IGF-1R pathway to develop its antitumor effect. A phase I pilot study designed to definitize maximum tolerated dose (MTD) and recommend phase II dose (RPTD) of AXL1717 adding to gemcitabine HCl and carboplatin finally established 215 mg BID as MTD and RPTD due to the bone marrow toxicities encountered in this trial (71). Besides, findings from an additional phase II randomized study, in which patients with advanced or metastatic NSCLC were treated with AXL1717 or docetaxel as a single agent, found no statistically significant between two treatment groups. The safety profile in this study concluded that there was a lower frequency of adverse events (AEs), especially neutropenia in the cohort of AXL1717 treatment. Unexpectedly, treatment-related fatal events were noted more commonly in this cohort (12 vs. 5) (72). On the whole, largely undesirable effects encountered in clinical settings emphasize the issue that identification of predictive markers indicative of responses to therapeutic strategies is timely and paramount to improving current therapy in carcinomas.

### Blocking Intracellular Signaling Pathways

With regard to a diverse array of intracellular signaling pathways resulting in EMT, inhibitors of these pathways are in active clinical development. On the basis of compelling preclinical evidence, RAS-RAF-MAPK pathway inhibitors have been put into clinical tests. A recent phase II non-randomized trial of combination BAF and MEK inhibition in patients with BRAF V600E-mutant NSCLC demonstrated a clear clinical benefit of dabrafenib plus trametinib, with a high overall response and manageable toxicity (73). Hampering the PI3k-AKT-mammalian target of rapamycin (mTOR) pathway is another means to prevent or reverse EMT. Pictilisib (GDC-0941), a pan-class PI3k inhibitor, had been reported to possess favorable safety and exert antineoplastic effects in solid tumors, including NSCLC, when administered in monotherapy or combination therapy as well (74–76). Furthermore, a recent phase I study of combination buparlisib (PI3k inhibitor) and radiotherapy in patients with NSCLC showed a well toleration and no dose-limiting toxicity (77). Beyond that, AKT inhibitor MK-2206 and mTOR inhibitor everolimus had been tested in the clinic reporting positive results through treatment for cancer (78, 79).

As mentioned before, key roles of Notch, Hedgehog and Wnt signal transduction pathways in EMT program and therapeutic resistance are widely embraced. Therefore, various of compounds targeting these pathways are now underway. For Notch cascade, classes of approaches that in clinical pipeline mainly compose of γ-secretase inhibitors (GSIs) and antibodies against the Notch receptor or ligand (30). At present, considerable clinical trials center on GSIs, which inhibit the generation of NICD to relay transcriptional signals subsequently. So far, a broad range of GSIs, known as PF-03084014, RO4929097, BMS-906024, and LY900009 are under preclinical or early clinical development in solid tumors inclusive of NSCLC (80–83). Besides using GSIs to interfere with the cleavage of Notch receptors, DLL4-specific mAbs inhibiting the ligand-receptor interaction reflects another viable option investigated clinically. A phase I FHD study examining enoticumab (REGN421) in eligible patients with advanced solid tumors, to determine the safety, toxicity and RPTD (84). Safety and efficacy of demcizumab in combination with standard chemotherapy was being assessed currently in NSCLC, implying the shorten treatment course in future development (85).

Abnormal activation of the Hedgehog signaling pathway is explicitly implicated in the biological behaviors of cancer, making targeted inhibition of this pathway a clinically useful therapeutic strategy. In this respect, a multiple of SMO inhibitors have entered clinical trials (86). As an example, a phase I study evaluating PF-04449913 in advanced solid malignancies showed prolonged stable disease in spite of no objective responses in this trial (87). TAK-441 has been also employed as a potential inhibitor in therapy with the effective outcomes of phase I trial (88). Of note, preliminary data from a study performed that investigated the safety of sonidegib (LDE225) in a total of 45 East Asian patients, suggested an ethnic difference in tolerability between populations (89). As with Notch and Hh pathway, there are intensive researches on targeting Wnt pathway for cancer therapeutics at the stage of preclinical and clinical testing, though no agents have been granted approval, thus far, to target such a pathway (90, 91). Recently, a comprehensive body of preclinical evidence that clofazimine specifically inhibits Wnt signaling pathway, without side effect, which possesses potential as a Wnt inhibitor (92).

IL-6 and STAT3 are frequently activated in carcinomas and drive the induction of EMT. An anti-IL-6 mAb, siltuximab, when used clinically to treat advanced solid malignancies, had been proven to be well-tolerated but without objective responses (93). STAT3 is a transcription factor downstream of IL-6 that indirectly induces EMT. A next-generation antisense oligonucleotide (ASO), termed AZD9150, displayed antitumor activity in lung cancer models and a phase I dose-escalation study involving patients with refractory NSCLC (94). Additional clinical trial with OPB-51602, a small-molecule STAT3-specific inhibitor, indicated most salient anticancer activity in NSCLC. Given its long half-life and poorer tolerability under continuous dosing, further research is warranted to exploit less frequent dosing schedule (95).

### Regulation of miRNAs and Epigenetics

MiRNAs regulate the EMT process by exciting or undermining EMT-related molecules. Even though a wealth of details is increasingly being recognized between miRNAs and EMT, to date, none of the EMT-associated miRNAs have been applied in clinical utility (44). With expanding knowledge of therapeutic miRNAs in recent years, it is legitimate to say that further work should concentrate on devising miRNA-targeting drugs as a strategy in cancer treatment (96, 97). Apart from the alterations in the genetic landscape, HDACs mediate epigenetic changes during EMT (98). This conceptual advance prompts HDAC inhibitors to translate to the clinic. Of 42 patients with NSCLC and head-and-neck cancer, incorporating an oral pan-HDAC inhibitor, termed as panobinostat (LBH589) with erlotinib potentiated the antitumor effect in a phase I trial (99). In combination with standard erlotinib, romidepsin achieved intriguing clinical activity in populations with NSCLC who did not respond well to EGFR-TKI monotherapy (100).

### Targeting CSCs

Cancer cells gain stem cell-like properties and present a picture of phenotypic diversity, partly brought out by EMT. For this reason, targeting EMT program offers a new ray of hope to eliminate CSCs and improve CSC-based therapy (6). The critical attributes of CSCs — stemness and EMT, contribute to the onset of resistance to therapeutic intervention (101). Cumulatively, treatment strategies operating to direct against CSCs include therapy targeting CSC-dependent signaling pathways, developing anti-EMT approaches and manipulating the tumor microenvironment (6, 51, 54). First, targeting CSCs via modification of CSC signaling, such as Notch, Hh, Wnt cascade, holds great promise of propelling the clinical update in cancer treatment (32). Second, CSC-targeted therapy can also be accomplished by thwarting EMT-related signaling pathways, given its interconnection with CSC biology (6). Finally, targeting the TME is an alternative possibility in the case of CSCs. As stated, diverse signals within TME can trigger the activation of EMT and entrance cancer cells into the CSC state, thus reducing the vulnerability to targeted agents (51, 54). Furthermore, therapeutic functions of miRNAs are coming into prominence for their multifaceted biology to control EMT and EMT-induced CSCs (102). Over the past few years, a renewed interest in immunotherapy for NSCLC treatment conduces to accelerate progress in targeting CSCs with immunotherapeutic methods (103). This knowledge necessitates rigorous clarification of the cellular and molecular mechanisms behind it to produce a durable response to therapy.

## Conclusions

EGFR as a therapeutic target, its role in patients with EGFR-mutant NSCLC is of the essence. Unfortunately, therapeutic susceptibility to EGFR-TKIs is affected by drug resistance (104). With a growing understanding of EMT, the research scope on it is expanding continuously. Most intriguing is how the EMT program gives birth to acquired resistance to EGFR-TKI-based treatment regimes. As argued above, EMT is a kind of highly sophisticated nonlinear dynamic process, which strongly linked with therapeutic responses, stated differently, crosstalk between aforementioned signaling pathways, subjected to genetic and epigenetic modifications, ultimately leading to resistance against anticancer therapy. The complexity and versatility of regulatory mechanisms exceed the expectation, bringing obvious challenges to devise anticancer therapies for clinical use. Another issue is to obtain a solid understanding of EMT-CSC link whereby we can strike at the root of evil (6).

Ongoing studies focusing on EMT make it lie at the heart of drug resistance in oncotherapy. Still, clinical practicable strategies to overcome resistance and improve survival are limited. Given that EMT is a highly regulated process in contextual settings and tightly associated with CSCs, from a therapeutic standpoint, a thorough grasp of mechanism basis is the determinant and first step in the long road fraught with difficulties. Moreover, immunotherapy opens up an attractive area of drug discovery for exploring and treating cancer, while the definition of potential biomarkers that correspond with treatment responses and the development of combinatorial strategies deserve deep concern in the future (105, 106). The personalized, precise diagnosis and treatment for NSCLC is currently thriving, it is safe to say, therapeutic approaches moving from bench to bedside to ensure optimal efficacy in cancer therapies are on the horizon (107).

## Author Contributions

XN conceived the project direction. XZ and LC inquired and collated all the literature. LC and LL read all the literature and wrote the manuscript. XN drawn the mechanism diagram.

### Conflict of Interest

The authors declare that the research was conducted in the absence of any commercial or financial relationships that could be construed as a potential conflict of interest.

## Abbreviations

EGFR-TKIs: epidermal growth factor receptor tyrosine kinase inhibitors
NSCLC: non-small-cell lung cancer
EMT: epithelial-mesenchymal transition
siRNA: small interfering RNA
ZEB: zinc-finger E-box-binding
TGF-β: transforming growth factor-β
Grb2: growth factor receptor-bound-2
HDAC6: histone deacetylase6
NICD: Notch intracellular domain
IGF: insulin-like growth factor
IGF-1R: insulin-like growth factor-1 receptor
IRS-1: insulin receptor substrate-1
MAML: Mastermind-like protein
Hh: Hedgehog
SHH: Sonic hedgehog
IHH: Indian hedgehog
DHH: Desert hedgehog
SMO: Smoothened
CSE: cigarette smoking extract
NAC: N-acetylcysteine
STAT3: signal transducer and activator of transcription 3
OSM: oncostatin-M
TEM: tumor microenvironment
JAK1: Janus Kinase1
miRNAs: microRNAs
3′-UTR: 3′-untranslated region
PTEN: phosphatase and tensin homolog
CSCs: Cancer stem-like cells
SMIs: Small molecule inhibitors
FHD: first-in-human dose
mAbs: monoclonal antibodies
SAEs: serious adverse events
OS: overall survival
MTD: maximum tolerated dose
AEs: adverse events
mTOR: mammalian target of rapamycin
ASO: antisense oligonucleotide.
